# R-loop and diseases: the cell cycle matters

**DOI:** 10.1186/s12943-024-02000-3

**Published:** 2024-04-27

**Authors:** Yuqin Xu, Yue Jiao, Chengbin Liu, Rui Miao, Chunyan Liu, Yilong Wang, Chunming Ma, Jiao Liu

**Affiliations:** School of Basic Medicine Sciences, Shandong Second Medical University, Weifang, 261053 China

**Keywords:** R-loop, Cell cycle, Transcription-replication conflicts, Genomic stability

## Abstract

The cell cycle is a crucial biological process that is involved in cell growth, development, and reproduction. It can be divided into G1, S, G2, and M phases, and each period is closely regulated to ensure the production of two similar daughter cells with the same genetic material. However, many obstacles influence the cell cycle, including the R-loop that is formed throughout this process. R-loop is a triple-stranded structure, composed of an RNA: DNA hybrid and a single DNA strand, which is ubiquitous in organisms from bacteria to mammals. The existence of the R-loop has important significance for the regulation of various physiological processes. However, aberrant accumulation of R-loop due to its limited resolving ability will be detrimental for cells. For example, DNA damage and genomic instability, caused by the R-loop, can activate checkpoints in the cell cycle, which in turn induce cell cycle arrest and cell death. At present, a growing number of factors have been proven to prevent or eliminate the accumulation of R-loop thereby avoiding DNA damage and mutations. Therefore, we need to gain detailed insight into the R-loop resolution factors at different stages of the cell cycle. In this review, we review the current knowledge of factors that play a role in resolving the R-loop at different stages of the cell cycle, as well as how mutations of these factors lead to the onset and progression of diseases.

## Introduction

All organisms on earth, from bacteria to humans, maintain their survival and keep the continuation of species through a constant repetition of cell growth and cell division. After a series of biochemical events, the components of cells are replicated and then equally divided into two parts to form two daughter cells. Cell division and correct separation of genomic materials between daughter cells are the basis of normal development, growth, and reproduction of organisms. The cell cycle is the mechanism of cell reproduction and is usually divided into four periods: G1, S, G2, and M. The DNA synthesis phase (S phase) and mitosis phase (M phase) are respectively separated by two-period gaps called G1 and G2 [1, 2]. The duration of the division phase accounts for a very small proportion of the whole cell cycle, and the overall time of the S, G2, and M phases is relatively constant. Therefore, the length of the cell cycle mainly depends on the G1 phase [3]. There is also a period in the cell cycle called the G0 phase in which cells are generally in a quiescent or dormant period. Some cells in G0 remain stationary for a long time until they are stimulated by external cues (i.e., growth factors) to re-enter the cell cycle and undergo cell division [4, 5]. Genome replication is a key step in the S phase. In this process, the replisome (DNA replication system) must overcome many obstacles, such as R-loop, that otherwise lead to replication fork stalling and genomic integrity impairment [6]. R-loop is a transcriptional complex consisting of a single-stranded DNA (ssDNA) and RNA: DNA hybrid produced during transcription [7]. Studies have shown that the R-loop exists independently of the replication process, and it formed at one stage of the cell cycle can be transmitted to another stage and the next cell cycle [8]. Therefore, the R-loop exists in all stages of the cell cycle and plays a crucial role in many physiological and pathological processes [9, 10]. The physiological role of the R-loop includes immunoglobulin (Ig) class switch recombination (CSR), gene expression, DNA replication, DNA repair, and regulation of transcription initiation/termination processes [11]. However, the temporal and/or spatial accumulation of unscheduled R-loop in the genome will play a detrimental function, such as transcriptional defects, transcription-replication conflicts (TRCs), cell cycle arrest, and genomic instability [12–16].

Recently, it is clear that the generation and resolution of the R-loop is regulated by different factors in different cell stages, and the coordination of these factors is very important for cells to regulate the homeostasis of the R-loop thereby protecting its genomes [8, 17–19]. These factors can cooperatively avoid the accumulation of the unscheduled R-loop by resolving it or even by preventing its initiation [11, 20]. It is worth noting that the presence of the pathological R-loop, caused by deficiencies or mutations in R-loop resolution factors, may hinder the process of the cell cycle [21]. Additionally, it is reported that the abnormal accumulation of R-loop can lead to diseases, such as neurological diseases and cancers [22]. Here, we review the relationship between the cell cycle and R-loop, the R-loop resolution factors acting on different stages of the cell cycle, and the diseases caused by the defective function of these factors.

## The cell cycle

**Fig. 1:**
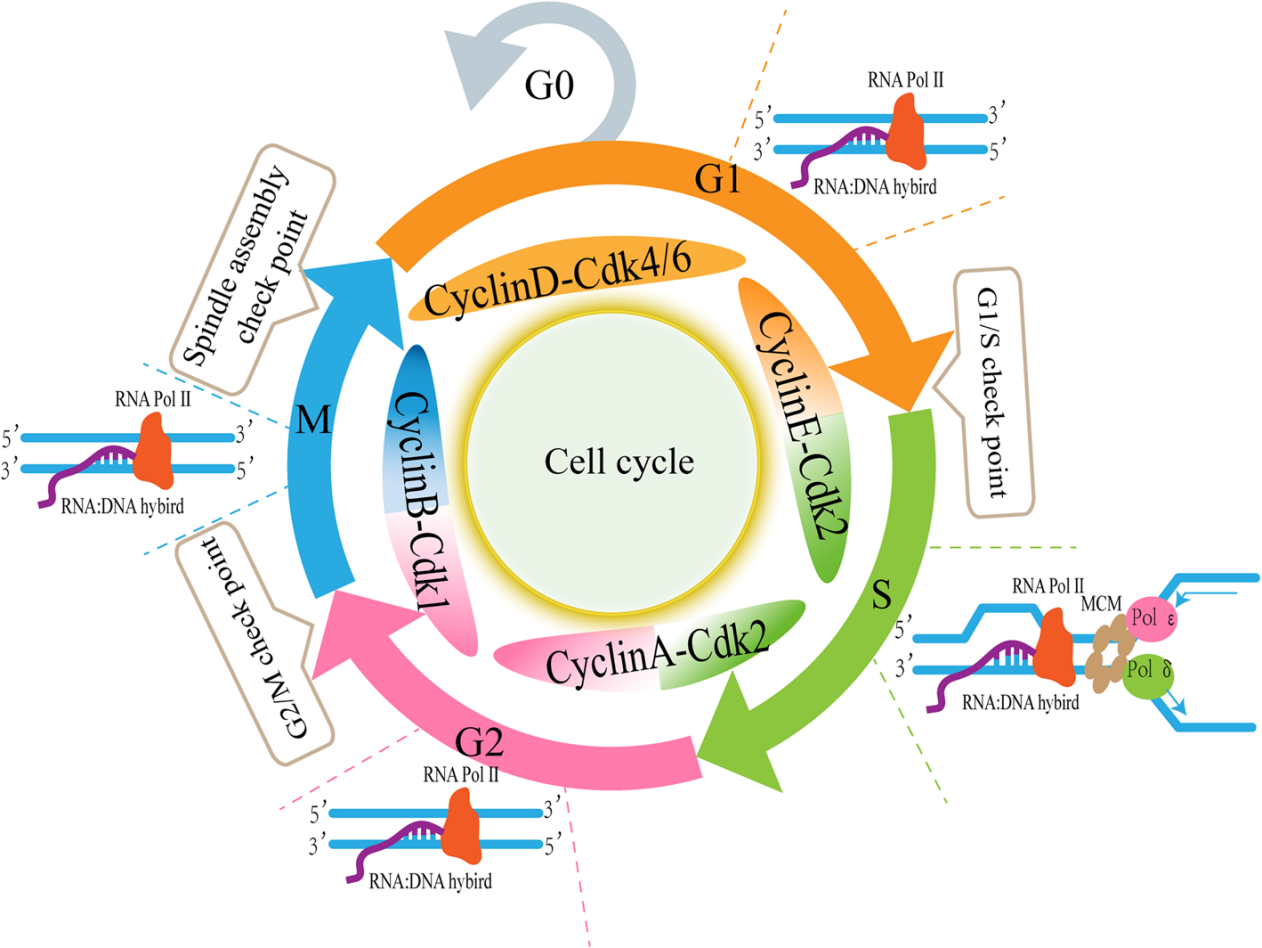
The cell cycle and check points. The cell cycle is an essential mechanism of cell proliferation, it can be divided into five stages: stationary phase (G0), pre-DNA synthesis (G1), DNA synthesis (S), post-DNA synthesis (G2), and mitosis (M). The whole cell cycle is regulated by a variety of cyclins and cyclin-dependent kinases (Cdks). In addition, G1/S check point, G2/M check point, and spindle check point play an important role in ensuring the normal progression of the cell cycle. R-loop can be formed spontaneously throughout the cell cycle and is most common during the S phase

The cell cycle is an extremely conserved process of life activity. As aforementioned, the cell cycle can be divided into G0, G1, S, G2, and M phases [23]. The S phase is the DNA synthesis phase, while the M phase can be further divided into two processes: nuclear division and cytoplasmic division. To ensure the accurate replication and separation of genetic material, cell cycle progression is tightly regulated by cyclin-dependent kinases (Cdks) and their regulatory cyclin subunits [1, 24, 25]. Different Cdks bind to their partner cyclins at different stages to properly order the events of the cell cycle [26, 27]. In the process of the cell cycle, there are three important checkpoints, including the G1/S checkpoint, G2/M checkpoint, and spindle assembly checkpoint (SAC), which serve as DNA surveillance mechanisms to prevent genetic errors during cell division [26, 28, 29] (Fig. 1). The G1/S checkpoint controls the entry of cells from the quiescent G1 to the DNA synthesis phase. The G2/M checkpoint is the control point that determines the cell division. SAC acts in the middle and late M phase to prevent chromosome separation until the sister chromatids are correctly connected to the mitotic spindle. Activation of checkpoints often results in cell cycle arrest, which provides a temporal delay to repair DNA damage [15].

## The generation of R-loop

**Fig. 2:**
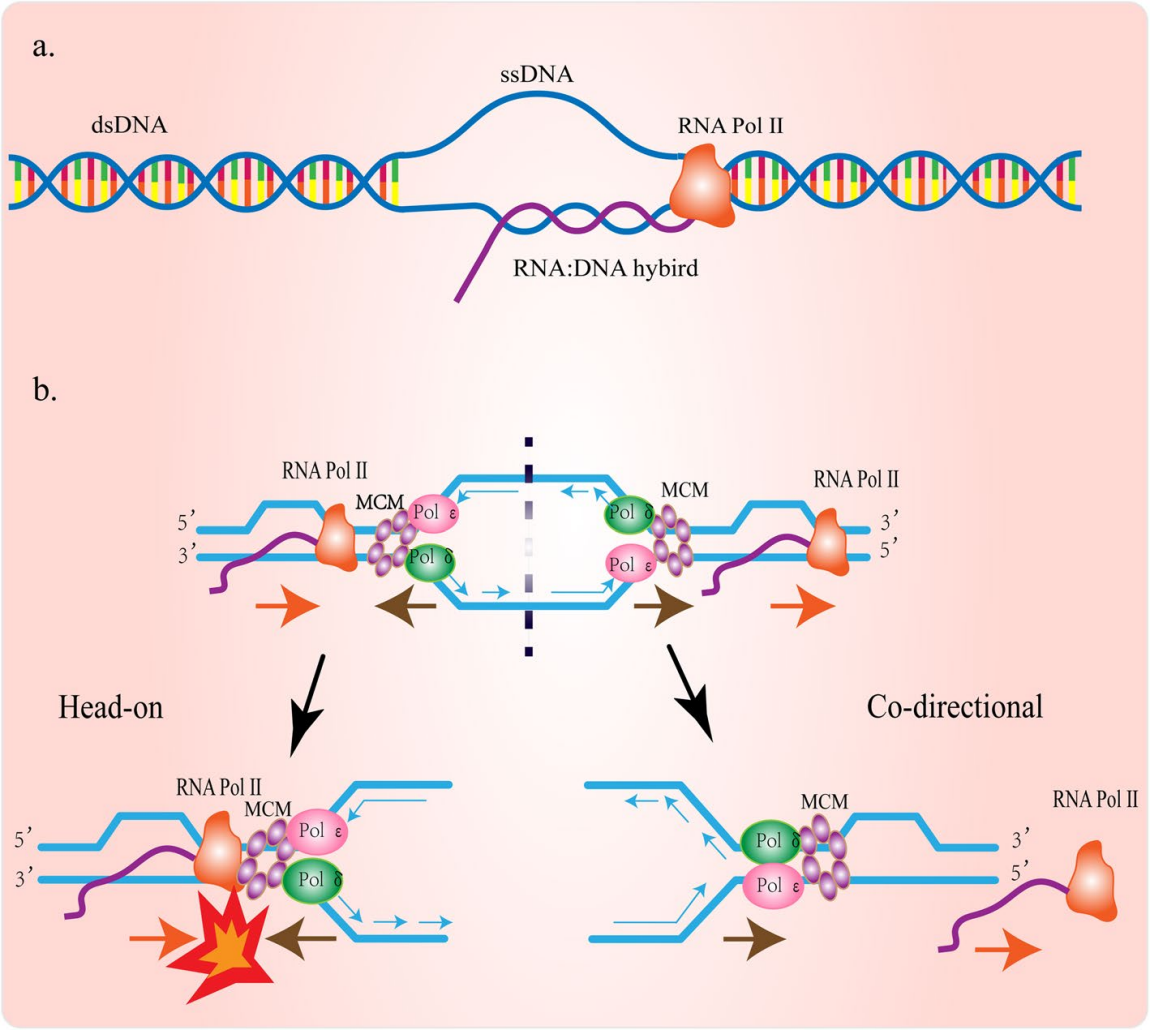
The structure of the R-loop and the process of transcription-replication conflicts. **a** R-loop is a three-stranded nucleic acid structure consisting of an RNA: DNA hybrid strand and a replaced DNA strand (ssDNA). **b** In the S phase of the cell cycle, transcription and replication share the same DNA strand as a template, which makes the two processes often collide. The transcription-replication conflict is divided into two types: HO and CD conflict. In the HO conflict, RNA polymerase II collides with the replicon, which leads to the generation of the R-loop. The consequences of co-directional conflict are different from those of HO conflict, which can avoid the accumulation of the R-loop

R-loop is a three-stranded nucleic acid structure formed during transcription, it is made up of a single-stranded DNA that has been displaced and an RNA: DNA hybrid [30–32] (Fig. 2a). In the process of transcription, the nascent RNA produced by RNA polymerase II(RNA Pol II) hybridizes with its template to form RNA: DNA hybrid strand [33]. R-loop length in highly transcribed genes can be greater than 1 kb [34]. R-loop can be mainly divided into two categories. One is the physiological R-loop, which plays an active role in many physiological activities. The other is the pathological R-loop which is formed in a non-procedural manner and poses a threat to the stability of the genome [11, 35]. Therefore, how is the R-loop generated? Under physiological conditions, a short RNA: DNA hybrid of about 8 bp is transiently produced by the binding of the newly synthesized RNA to the template DNA during transcription. Then, a special protein moiety of RNA polymerases (RNAPs) opens a channel for RNA to be stripped from the DNA template chain to ensure that the RNA: DNA hybrid produced during this process does not accumulate [36, 37]. Under pathological conditions, such as topoisomerase flaws, and RNA damage [38], R-loop can be abnormally formed and accumulated. The majority of unmethylated CpG islands (CGIs) promoters in the human genome have a large amount of guanine and cytosine chain asymmetry, which is called GC skew. Due to the greater thermal stability of GCs, the GC skew confers significant potential to form the R-loop structure when the newly transcribed G-rich RNA strand is annealed back to the template C-rich DNA strand [39, 40].

The repeat sequences in the genome are also easy to form R-loop structures. These repeat sequences are called microsatellites or short tandem repeats and have the potential to generate secondary structures, including hairpins, cruciform, triplex DNA, and G-quadruplexes (G4), these structures might obstruct DNA replication [41, 42]. Studies have shown that a variety of disease-related trinucleotide repeats, such as FRAXA(CGG)·(CCG) repeats, SCA1(CAG)·(CTG) repeats, DM1(CTG)·(CAG) repeats and FRDA(GAA) repeats, can form stable R-loop in vitro by transcription induction. The formation and maintenance of the R-loop require a negative supercoiled template [43]. The human fragile X mental retardation 1 gene (FMR1) contains a (CGG)_n_ trinucleotide repeat. The transcription of the GC-rich FMR1 5'UTR region is conducive to the formation of R-loop. In addition, the amplification of CGG repeats and related transcription lead to increased formation of R-loop, which are more likely to fold into complex secondary structures [44].

When the R-loop is excessively accumulated or present in the wrong position in the genome, it poses a threat to the stability of the genome [12]. The generation of pathological R-loop is generally due to the deficiencies or mutations of important proteins involved in life activities.

## The function of R-loop

The R-loop, which is present in all organisms, plays a double-edged sword role. On the one hand, the R-loop is crucial for several physiological processes, such as chromosome separation during mitosis, CRISPR-Cas9 bacterial defense, immunoglobulin (lg) switching, DNA replication and repair, and regulation of transcription initiation/termination processes [45, 46]. On the other hand, when the unplanned R-loop is generated and accumulated, it causes a series of damage reactions, including DNA double-strand breaks and genomic instability, which further lead to hypermutation, cell cycle arrest, and even cell death [47].

For example, telomeres are nucleoprotein structures at the ends of linear chromosomes in heterochromatin regions [48]. Telomeric repeat-containing RNA (TERRA) is a long noncoding RNA, it forms physiologically relevant RNA: DNA hybrids at telomeres [49]. Telomere shortening caused by a lack of telomerase activity can lead to premature senescence, while the presence of R-loop on short telomeres helps to activate DNA damage response (DDR) and promote the recruitment of Rad51 recombinase, thereby preventing early senescence onset [50]. However, in Saccharomyces cerevisiae, the accumulation of R-loop caused by hpr1 mutations leads to DNA breaks and activation of DNA damage checkpoints, which in turn leads to defective meiosis [10].

## Multiple types of factors involved in R-loop regulation

Because RNA: DNA hybrids are important sources of structure changes in the genome, therefore, preventing the formation and accumulation of pathological R-loop has become an effective way to maintain genomic stability. In this process, RNase H and a variety of RNA biogenesis factors act as guardians of the genome's genomic stability [51]. To reduce DNA damage caused by the accumulation of R-loop, organisms can avoid the accumulation of R-loop by preventing its formation. Alternatively, it can also resolve the formed R-loop by using multiple types of factors [11]. So far, many factors that play roles in R-loop resolution have been found. These factors include ribonuclease, helicase, topoisomerase, DNA damage repair factors, transcription and RNA processing factors, chromosome remodeling factors, and RNA modifiers (Table 1). Specifically, ribonuclease is an enzyme that can degrade the RNA portion of the RNA: DNA hybrids. So far, there are two common ribonucleases, RNase H1 and RNase H2 [52, 53]. RNA helicase has RNA: DNA unwinding activity in vitro and can unwind RNA: DNA hybrids, such as DDX19, UAP56, and DDX1 [54]. DNA topoisomerases play an important role in R-loop prevention. The function of these enzymes is to solve the torsional stress during transcription and replication. Topoisomerase 1 (Top1) and Topoisomerase 2 (Top2) can relax the positive and negative supercoils in DNA [55]. Top1 is an enzyme that relaxes DNA supercoils and prevents R-loop formation, which makes it an important player in regulating R-loop homeostasis by ensuring faithful replication and genomic integrity [56]. When Top1 is deficient, R-loop-driven replication stress occurs, which leads to DNA damage [57]. Many factors involved in the repair of DNA damage also play important roles in the resolution of R-loop, such as BRCA1, BRCA2, and Fanconi anemia factor [58]. BRCA1 regulates the G2/M checkpoint by activating Chk1 kinase during DNA damage [59]. It has been reported that BRCA1 can form a complex with Senataxin (SETX) and deal with R-loop-related genomic instability at transcriptional termination pause sites [60]. In addition, many molecules in the DEAD-box (DDX) family have also been reported to resolve the R-loop. At present, more than 35 DDX helicase members have been found in humans, and these members are highly conserved [61]. The THO complex is a common transcription and RNA processing factor that has important roles in transcription elongation, RNA processing and export. Inactivation of the THO complex can cause R-loop accumulation which further leads to genomic instability [62]. At the same time, some chromatin factors are also important participants in R-loop homeostasis maintenance and genomic stability. Studies have shown that chromosome remodeling complexes help to resolve R-loop-mediated TRCs. SWI/SNF, ISWI, CHD, and INO80 are four major chromatin remodeling families [63, 64]. In addition, m6A RNA methylation modification is involved in the regulation of R-loop level [65, 66]. Another enzyme, Flap endonuclease 1 (FEN1), plays an important role in the maintenance of genomic stability by using its nucleolytic activity to resolve the R-loop [67]. Additionally, studies have shown that sterile alpha motif and HD domain-containing protein 1 (SAMHD1) and RNA-specific adenosine deaminase 1(ADAR1) can play the role of resolving the R-loop, thereby avoiding the threat to the genome [68–70].

**Table 1:** Factors regulating R-loop homeostasis

Type of factors	Factors	Function	Reference
Ribonucleases	RNase H1, RNase H2 [19]	resolution	[19]
Helicases	C1orf109L [18], UAP56/ DDX39B [54], FEN1 [67], DDX18 [71], SETX/Sen1 [72], DDX17 [73], DDX19 [74], DDX5 [75], DDX1 [76], DDX41 [77],DDX21 [78], AQR [79], BLM [80]	resolution	[18, 54, 67, 71–80]
	DHX9 [38]	formation	[38]
Topoisomerases	Top1 [56], TOP2A [81]	resolution	[56, 81]
DNA damage repair factors	MUS8 [12], BRCA1 [60], SAMHD1 [68], PrimPol [82], BRCA2 [83], MRE11 [84], Fanconi anemia factor, XPG, XPF, XAB2 [85], CtIP [86], APTX, TDP1 [87]	resolution	[12, 60, 68, 82–87]
	RAD51 [88], RPA [89]	formation	[88, 89]
Transcription and RNA processing factors	Thrap3 [45], THO complex [62], TonEBP [65], SPT6 [90], WDR33 [91], XRN2 [92]	resolution	[45, 62, 65, 69, 90–92]
	CE (capping enzyme) [93]	formation	[93]
Chromosome remodeling factors	SIN3A [17], BRD4 [46], SWI/SNF [64], INO80 [94], SIRT7, PBRM1, ATRX [81], FACT [95], BRD2 [96]	resolution	[17, 46, 64, 81, 94–96]
RNA modification (Writers & Readers)	METTL3 [65], YTHDF2 [66], ADAR1 [69], TRDMT1 [81]	resolution	[65, 66, 69, 81]

The reproduction of all organisms relies on the cell cycle. In this process, there are strict regulatory mechanisms at each stage to ensure the smooth progress of the cell cycle, but the prevalent emergence of unplanned R-loops makes it bumpy. Therefore, it is particularly important to understand which factors play their roles in resolving the R-loop at a specific stage of the cell cycle.

## The relationship between the cell cycle and R-loop

### Formation of R-loops during S phase due to transcriptional replication conflicts

Studies have shown that the R-loop can be formed spontaneously throughout the cell cycle and is the source of DNA damage. S9.6 antibody is a widely used tool for purification, analysis and quantification of R-loop structure. It binds to RNA: DNA hybrids in a sequence-independent manner and has high affinity for RNA: DNA hybrids [97]. By using flow cytometry, Sonia Barroso et al. measured the S9.6 Immunofluorescence (IF) intensity of whole cells pretreated with RNase III to analyze the level of RNA: DNA hybrids at different cell cycle stages, they found that the fluorescence intensity was increased significantly from G1 to G2, these results suggest that the de novo formation of R-loop occur from G1 to G2 [98].

Replication and transcription are normally carried out independently during the S phase of the cell cycle [99]. However, under some conditions, transcriptional replication conflicts (TRCs) often occur due to the two processes share the same template. TRCs can occur when replication and transcription machinery encounter in a head-on (HO) orientation, they move toward each other, or in co-directional (CD) orientation, they move in the same direction [100] (Fig. 2b). The S phase of eukaryotic cells is the most fragile period in which replication and transcription coexist temporally and spatially, so TRCs take place during the S phase [16]. Transcription is thought to go more quickly than replication. In particular, the transcription rate of RNA pol II in mammalian cells is roughly 3.8 kb/min, whereas the average replication rate in human cells is 1.5-2 kb/min [101]. TRCs may result in replication fork blocking, premature termination of transcription, DNA damage, and recombinant intermediates, which put the integrity of the genome in danger. Further study demonstrates that the level of the R-loop depends on the direction of the TRCs. HO collisions of TRCs aggravate the production of the R-loop, whereas CD collisions avoid the accumulation of the R-loop. The explanation is that HO collisions, rather than CD collisions, may block transcription, thereby confining nascent RNA strands near the DNA template and promoting the formation of RNA: DNA hybrids [102, 103]. Furthermore, HO collisions can lead to a pause in the replication process and a high level of hyper-recombination, while CD collisions will not lead to such a consequence. The difference between them may be due to the termination of transcription by RNA polymerase after TRCs, compared with head-on encounters, co-directional encounters can be avoided to some extent [104]. For bacteria and eukaryotes, the degree of topological complexity caused by TRCs is different. Bacteria have only one replication starting point, and the direction of transcription and replication conflicts is mostly CD collisions [105]. In eukaryotes, chromosome replication is initiated from multiple replication origins, and TRCs often occur as HO collisions, increasing the complexity of the topology [106]. Therefore, the genomic integrity of eukaryotic cells is easily threatened by the conflict between transcription and replication.

### Activation of cell-cycle checkpoints by R-loops

**Fig. 3:**
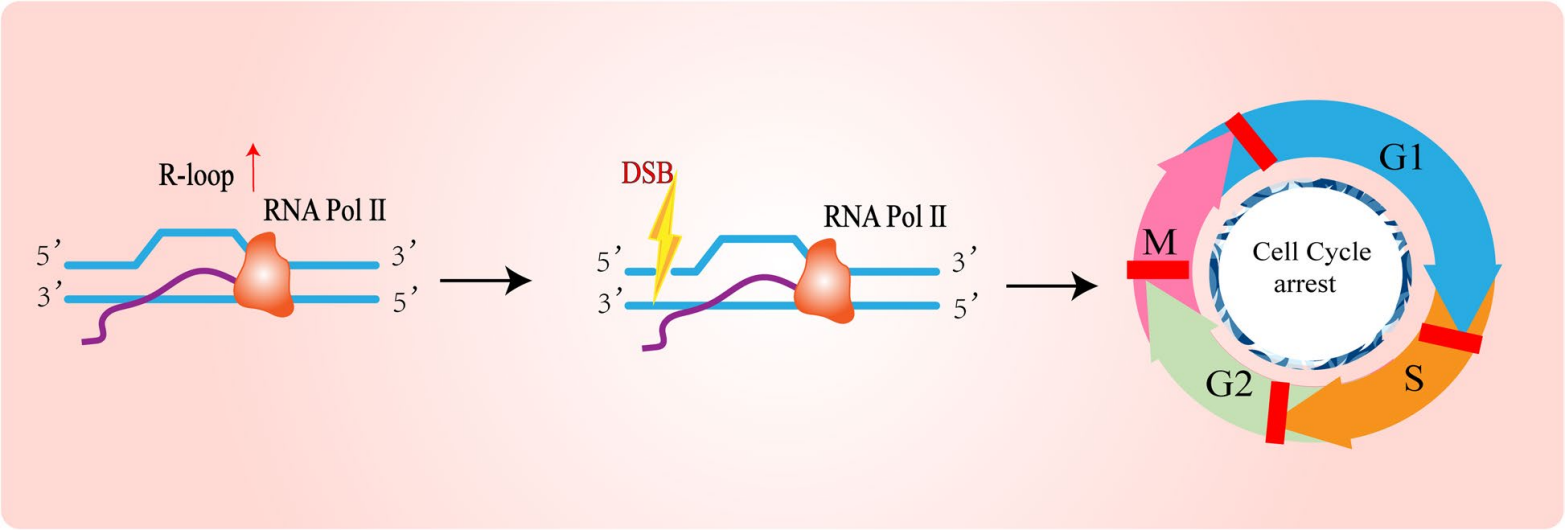
The increase of R-loop level leads to DNA double-strand breaks and cell cycle arrest. When cells encounter replication stress and other pressures, the existence of checkpoints can block the transition between different stages of the cell cycle and avoid the threat of the genome. R-loop-mediated DNA damage triggers the activation of cell cycle checkpoints, which are indispensable for cellular survival during replication stress

In addition, there are some important checkpoints in the cell cycle which include G1/S, G2/M, and SAC checkpoints. When cells encounter replication stress and other pressures, the existence of checkpoints can block the transition between different stages of the cell cycle and avoid the threat of the genome. For example, when replication is abruptly halted, the checkpoints of the cell cycle can tightly control the stability of stalled forks [107]. When DNA is damaged, it activates checkpoints to delay cell cycle progression and DNA damage repair. In general, the activation of DNA damage checkpoints means cell cycle arrests (Fig. 3). When G1 and G2 checkpoints are activated, the cell will initiate a mechanism to suspend the cell cycle. The response of cells in the S phase to DNA damage is completely different from that of the previous two phases. It does not induce an immediate cell cycle arrest but rather decelerates progression before entering the subsequent stage [15]. Additionally, R-loop-mediated DNA damage triggers the activation of cell cycle checkpoints, which are indispensable for cellular survival during replication stress [108]. Studies have shown that the mcm2DENQ (Alleles of Mcm2-7, the catalytic core of the replicative helicase and a part of DNA replication checkpoint signaling cascade) mutation leads to the formation of RNA: DNA hybrids in the S phase, which will persist until the cell pass the spindle assembly checkpoint. These results indicate that the Mcm replicon helicase can prevent the accumulation of RNA: DNA hybrids and it also plays a crucial role in avoiding the conflict between transcription and replication [109, 110].

### Factors that resolve the R-loop in the G1/S phase

**Fig. 4:**
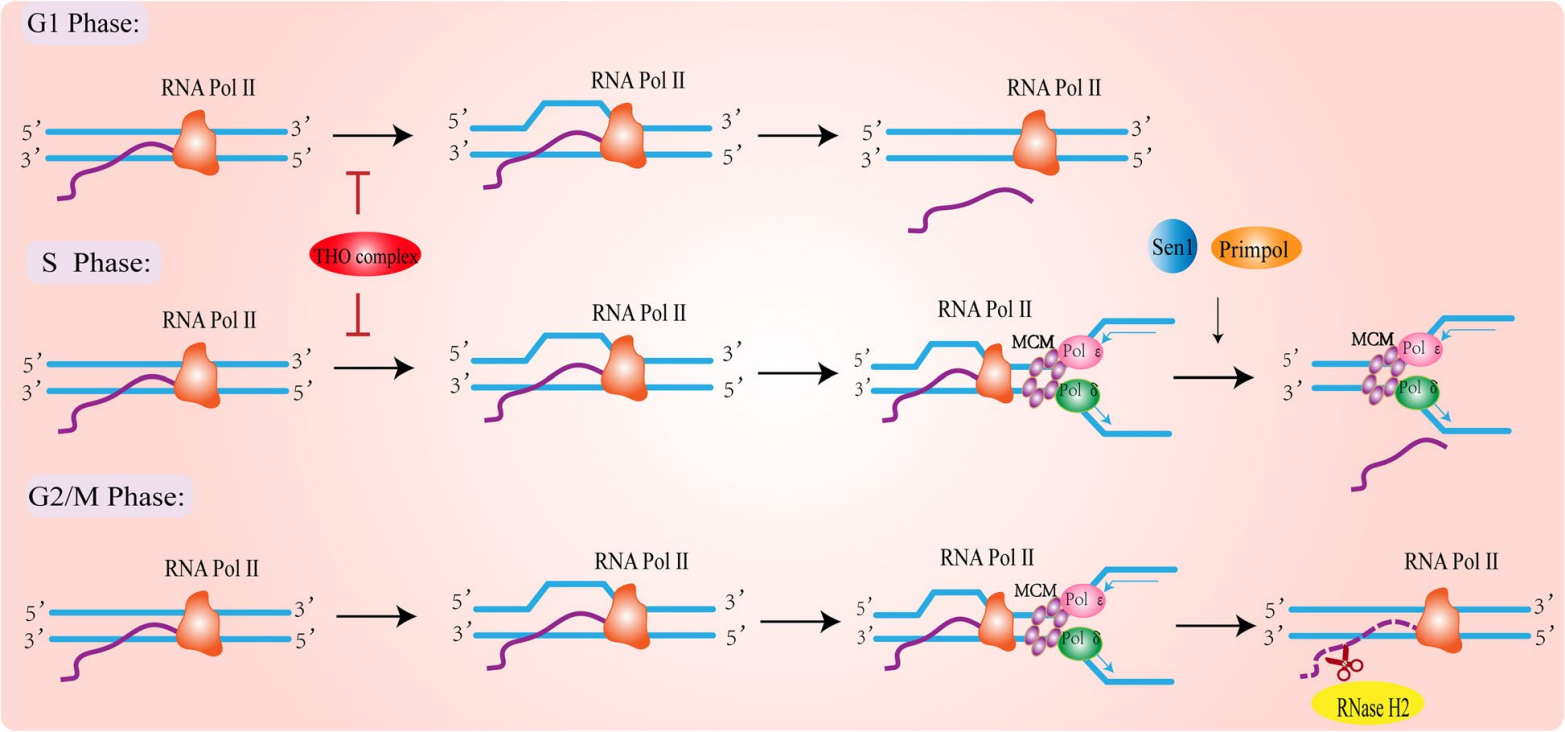
Cell-cycle-dependent R-loop resolution factor. For the cell-cycle-dependent R-loop factor, the THO complex plays a role in both the G1 phase and the S phase. In addition, Sen1 and Primpol can also resolve it in the S phase. In the G2/M phase, RNase H2 is the principal factor that resolves the RNA portion of the RNA: DNA hybrid strand.

The decision made by cells to transition from the G1 phase of the cell cycle to the S phase is crucial for normal development. During the G1 phase, cells decide whether to enter into the cell cycle, initiating DNA replication and division, or exit from it and enter stasis, senescence, or differentiation [111]. The maintenance of genomic integrity and the stable transmission of genetic information depend on many DNA repair processes. Failure to faithfully carry out these processes can lead to genetic material mutations and the development of genetic diseases [112]. In addition to DNA damage repair factors, RNA binding factors also play a key role in preventing genomic instability caused by the accumulation of the R-loop. The common RNA binding factor is the THO complex, and other factors related to RNA processing have similar functions in avoiding the accumulation of the R-loop, such as SETX/Sen1, DDX19, or DDX23 [17]. A recent study found that the THO complex can effectively prevent the accumulation of R-loop in the G1 and S phases. The factors involved in the resolution of the R-loop in the S phase are SETX/Sen1 and primase–polymerase (PrimPol) [8, 82] (Fig. 4)

The THO complex is a conserved eukaryotic complex that acts on transcriptional elongation and RNA processing and export. The THO complex is very important for the formation of optimal messenger ribonucleoproteins (mRNPs) during transcriptional elongation. By ensuring the optimal packaging of mRNPs and by interacting with histone deacetylases ( such as Sin3A ), the THO complex may facilitate the transient closure of chromatin, thereby preventing the formation of R-loop [62].This protein complex consists of five interacting subunits in yeast, namely: Tho2, Hpr1, Mft1, Thp2, and Tex1 [113]. It has been found that the complex contains six members (THOC1-THOC6) in higher organisms [114]. When Hpr1, one of the subunits of the THO complex, was knocked down, the R-loop was greatly accumulated in G1-arrested cells or after entering the S-phase, which suggests that the THO complex could prevent the production of the R-loop in the G1 and S phase [8]. Deletion of THOC1 also leads to R-loop-dependent genomic instability [115]. It is reported that transcription increases mutation and recombination in bacteria, yeast, and humans [116]. The recombination caused by transcription mainly occurs in the S phase, which is related to the replication fork damage caused by TRCs [117]. The replication fork is a relatively fragile structure that often encounters obstacles that cause it to pause [118]. The increase in the co-transcriptional R-loop caused by the THO mutant is one of the factors that hinder the progress of the replication fork [8]. R-loop accumulation produces excessive ssDNA that triggers the activation of the S phase checkpoint. In summary, R-loop-mediated DNA damage caused by THO mutants can activate the S phase checkpoint, which is necessary for maintaining the stability of genetic material under replication stress [108].

In the past few years, many RNA helicases have been reported to be involved in the dynamics and stability of the R-loop. SETX is one of the earliest reported compounds. It is a human homolog of yeast Sen1p and is involved in RNA maturation and termination [119]. SETX is an RNA/DNA helicase that is considered to be involved in transcription and genome integrity maintenance. It is highly conserved throughout evolution and involves various biological processes from transcription termination to meiosis completion and genome integrity maintenance [120]. Sen1 helicase plays important roles in transcription termination and maintaining genomic stability. It has been found that SETX retains the function of its yeast homolog in transcription termination, but also acquires specific properties or characteristics of mammalian RNAPII [121]. The R-loop formed at the G-rich pause site, broken down by SETX, is a key step in the transcription termination [122]. Studies have demonstrated that the protein expression of Sen1 is subject to regulation by the cell cycle. By comparing the level of Sen1 at different stages of the entire cell cycle, Mischo et al. observed that its expression increased in the S and G2 phases. They posit that this phenomenon occurs due to an adaptive adjustment in Sen1 levels based on cellular requirements. During certain moments in the cell cycle, such as transcription encounters replication in the S phase, polymerase II is suspended and there is an increased likelihood of R-loop formation. Consequently, a higher level of Sen1 is required to fulfill its role in resolving the R-loop and maintaining genome stability [123, 124]. SETX can resolve RNA: DNA hybrids, its lack causes the accumulation of R-loop and DNA double-strand breaks, which in turn leads to genomic instability [125]. The conserved Sen1 helicase not only terminates non-coding transcription, but also interacts with replicators, and is reported to solve the genotoxic R-loop [126]. Martin-Alonso et al. found that the THO transcription complex can prevent the formation of the R-loop in both the G1 and S phases, while Sen1 RNA /DNA helicase can only specifically prevent the formation of the R-loop in the S phase of the cell cycle [8].

Additionally, primer polymerase (PrimPol) is also an important player during the process of R-loop resolution. Studies have shown that purine-rich repeats (GAA)_10_, G-quadruplex and H-DNA motifs containing secondary structure-forming sequences can hinder the process of replication, which is mainly attributed to the formation of RNA: DNA hybrids. The replication of these sequences requires the participation of PrimPol. The miss of PrimPol leads to an increase in the level of unscheduled R-loop around these sequences and becomes a replication barrier. Thus, PrimPol can use its reprime function to prevent excessive single-stranded DNA exposure to the S phase, thereby limiting the formation of the R-loop in the S phase [82].

### Factor that resolve the R-loop in the G2/M phase

At present, most of the studies on the R-loop rely on the S9.6 antibody. To test the specificity of this antibody, researchers usually carry out RNase H treatment, which can cleave RNA in RNA: DNA hybrids. When RNase H is added, the accumulation of R-loop will be greatly reduced [127]. RNase H can be divided into two types: RNase H1 and RNase H2. The points shared by them are that they localize in the nucleus and function to reduce the accumulation of R-loop in the genome by resolving RNA: DNA hybrids formed during transcription. In addition, RNase H2 also plays a role in ribonucleotide excision repair (RER) [128]. In yeast, RNase H1 is encoded by RNH1 and RNase H2 is encoded by RNH201 [129]. The structure of the two RNase H enzymes is different, RNase H2 is a heterotrimeric enzyme, while RNase H1 is a monomeric structure. Compared with the monomer, it is more difficult for the polymer to exert its function. Studies have shown that both the mRNA expression levels and activities are different between the two enzymes throughout the cell cycle. The mRNA level and enzyme activity of RNase H1 are constant in the cell cycle, while for that of RNase H2, there are two peaks in the S phase and G2 / M phase, respectively. Therefore, RNase H1 is often used as a means to detect the specificity of the S9.6 antibody in the current research on the R-loop [19, 54, 71, 88, 94, 130]. The activity of RNase H2 increases when the RNH1 gene is deleted, suggesting that it has a complementary effect involved in the resolution of RNA: DNA hybrids [131]. A recent study showed that RNase H1 and RNase H2 are different in the regulation of RNA: DNA hybrids in different phases of the cell cycle. RNase H1 plays a role in RNA: DNA hybrids at all phases of the cell cycle, while RNase H2 exerts its function only at specific G2/M. The functional differences can also be attributed to the different chromatin association between the two enzymes. When the cells enter the S phase and G2 / M phase, the affinity between the RNaseH2 subunit and chromatin increases, while the RNaseh1 subunit is weakly associated with chromatin throughout the cell cycle. In addition, RNaseH1, can serve as an excellent stress sensor and respond to stress caused by R-loop accumulation at any stage of the cell cycle [19] (Fig. 4). Also, in telomeres, RNase H2 interacts with the telomere binding factor Rif2 and is recruited into telomeres to degrade TERRA R-loop in the late S phase, which is coordinated with the telomere replication process [50].

Moreover, Zimmer et al. demonstrated that the genome regions protected by RNase H1 and RNase H2 from R-loop-mediated damage are different. As two evolutionarily conserved enzymes, RNase H2 has a global function in preventing chromosome instability caused by RNA: DNA hybridization. In contrast, RNase H1 has a region-specific function [132]. Taken together, there are significant spatio-temporal differences between RNase H1 and RNase H2 in R-loop resolution.

### Factors that resolve the R-loop throughout the cell cycle

**Fig. 5:**
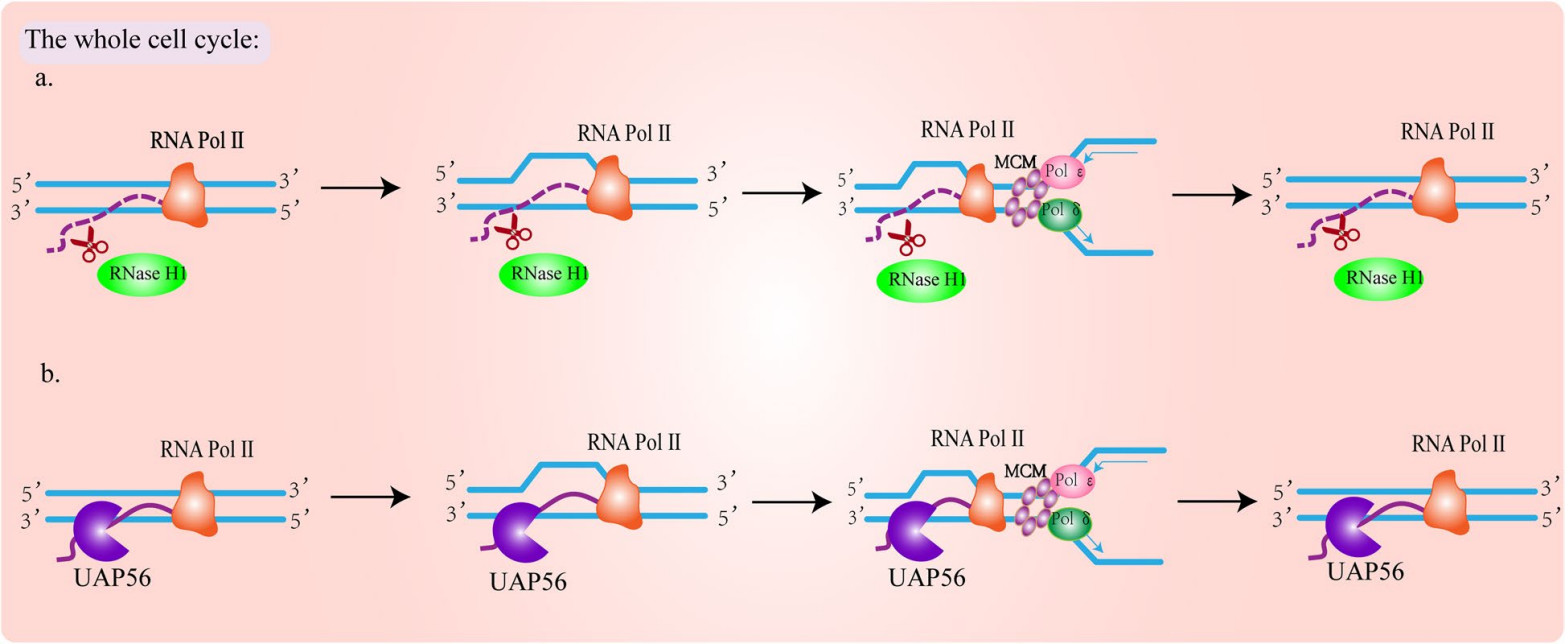
Cell-cycle-independent R-loop resolution factor. **a** Regardless of the cell cycle status, RNase H1 can reduce the accumulation of the R-loop by cutting the RNA portion of the hybrid, which is attributed to the constant high expression level of RNase H1 in the cell cycle. **b** UAP56/DDX39B is an effective helicase that prevents and/or eliminates the co-transcribed R-loop. Its role is to release new RNA from DNA, giving it the opportunity for further processing

The R-loop is ubiquitous throughout the cell cycle, that is G1, S, G2, and M phases. In addition to the above-mentioned factors that prevent the R-loop from producing or promoting the resolution of the produced R-loop at a specific period, some other factors are expressed and exert their functions in a cell cycle-independent manner. For example: RNase H1, UAP56/DDX39B [19, 62] (Fig. 5a, b).

It has been shown that RNase H1 is very important in the movement of the replication fork by resolving the R-loop. When RNH1 is depleted, it can lead to the accumulation of RNA: DNA hybrids, slowing down the movement of replication forks, and increasing the DNA damage [133]. RNase H1 is considered to be a stress sensor to responds to the R-loop accumulation when it reaches a toxic level regardless of the cell cycle. Nguyen et al. showed that replication protein A (RPA) can recognize ssDNA and interact with RNase H1, enhancing the binding of RNase H1 to RNA: DNA hybrids, and stimulating the activity of RNase H1 [134]. Another factor, UAP56/DDX39B is a partner of the THO complex and is involved in preventing the accumulation of R-loop induced by transcription [62]. It is demonstrated that UAP56 has strong RNA-DNA helicase activity, which can separate RNA: DNA hybrids and release completely new RNA molecules to ensure the smooth progress of RNA processing and output [54]. Pérez-Calero et al. found that the accumulation of R-loop and DNA damage was detected throughout the cell cycle after UAP56 depletion. And overexpression of wild-type UAP56 rescued the accumulation of RNA: DNA hybrids and R-loop-related genomic instability. This indicates that UAP56 regulates the homeostasis of the R-loop throughout the cell cycle. Furthermore, by measuring the distribution of RNA: DNA hybrids after UAP56 deletion, the author found that the R-loop was accumulated in the promoter region of the antisense RNA, throughout the gene body and the transcriptional termination region, it confirmed the global function of UAP56 in preventing R-loop accumulation [54, 135].

### Relationship between R-loop and diseases

As aforementioned, the R-loop plays an important role in many physiological processes, its aberrant presence in the genome is associated with several diseases. It is reported that some trinucleotide repeat-associated diseases, neurological diseases, and cancers are related to the R-loop [136](Table 2). Many proteins with proper function are essential for preventing DNA damage caused by the R-loop and genomic instability. Once these proteins are mutant or deficient, they can lead to the perturbation of R-loop homeostasis and the occurrence of diseases.

**Table 2:** R-loop and Links to Human Disease

Disease	R-loop factors	Suggested mechanism	References
Ovarian cancer	ADAR1	Silence of ADAR1 repressed ovarian cancer cell growth and caused R-loop abnormal accumulation.	[70]
Breast and ovarian cancers	BRCA1 BRCA2	The mutations or deletions of BRCA1 and BRCA2 can lead to increased R-loop levels and DNA damage.	[60, 137, 138]
Aicardi-Goutières syndrome (AGS)	TREX1 RNase H2 SAMHD1	Mutations in TREX1, RNASEH2A, RNASEH2B, and SAMHD1 are associated with the accumulation of RNA: DNA hybrids over repeat-rich intergenic and gene body regions.	[139]
		R-loops are highly enriched at transcription-replication conflict regions of the genome in fibroblast patients bearing SAMHD1 mutation.	[68]
Type 4 amyotrophic lateral sclerosis (ALS4)	SETX ZPR1	ZPR1-deficiency causes downregulation of SETX and accumulation of R-loops.	[140]
Amyotrophic lateral sclerosis (ALS)	TDP-43	Mutated TDP-43 in transfected neuronal SH-SY5Y and lymphoblastoid cell lines (LCLs) from an ALS patient cause R-loop accumulation.	[141]
Ataxia with oculomotor apraxia 2 (AOA-2)	SETX	SETX can resolve the R-loop in AOA2 iPSCs.	[142]
Hepatocellular carcinoma (HCC)	THOC1 MTA2 TonEBP	Knockdown of THOC1 leads to R-loop formation, and DNA damage and confers sensitivity to cisplatin.	[143–145]
		MTA2 could interact with HDAC2/CHD4, and transcriptionally inhibit BDH1 by R-loop, leading to HCC formation and progression.	
		TonEBP prevents R-loop-mediated DNA damage in HepG2 human hepatoma cells.	
Testicular seminoma (TS)	BRE1	Aberrant R-loop constitutes a significant source of DSBs in BRE1-deficient cells.	[146]
Amyotrophic lateral sclerosis (ALS) and frontotemporal dementia (FTD)	C9orf72	C9orf72 repeat expansion can induce DNA damage response leading to ALS/FTD pathologies.	[147]
Friedreich ataxia (FRDA) and fragile X syndrome (FXS)	FXN FMR1	R-loop acts as an initial trigger to promote FXN and FMR1 silencing.	[148]
Xeroderma pigmentosum (XP)	XPF XPG	Processes R-loops to limit their levels.	[85, 149]
Kaposi’s Sarcoma (KS)	THO/TREX complex	THO/TREX prevents the formation of R-loop that can compromise genome integrity.	[115]
Fanconi anemia (FA)	FANCM FANCD2	Suppresses R-loop and prevents R-loop-dependent DNA damage.	[58]
Spinal muscular atrophy (SMA)	SMN1 ZPR1	ZPR1 rescues defective RLRC assembly and prevents pathogenic R-loop accumulation in SMA.	[140]
Multiple cancers	INO80 HRAS	INO80-dependent resolution of R-loop promotes DNA replication.	[57, 94]
		Mutated HRAS activation causes aberrant replication fork acceleration and DNA damage by decreasing the R-loop.	
Myelodysplastic syndromes (MDS)	DDX41	DDX41 suppression of R-loop levels and inflammatory signaling.	[150]
Immunodeficiency, centromere instability, and facial anomalies syndrome (ICF)	TERRA	RNA: DNA hybrids promote damage and instability at telomeric regions in ICF.	[151]

Trinucleotide repeats are easy to form RNA: DNA hybrids in vivo, and the presence of multiple repeats results in human diseases. For example, CGG repeat amplification is associated with type 1 myotonic dystrophy (DM1), type 2 myotonic dystrophy (DM2), spinocerebellar ataxia type 8 (SCA8), and other diseases [152].

Aicardi-Goutières syndrome (AGS) is an autosomal recessive genetic disease with typical clinical manifestations of neurological dysfunction. It has been reported that AGS is caused by mutations in genes encoding intracellular nucleic acid metabolic enzymes, including 3’→5’ DNA exonuclease (TREX1), RNase H2, SAMHD1, and ADAR1 [153]. In humans, the RNase H2 catalytic subunits RNASEH2A, RNASEH2B, and RNASEH2C are all required for enzymatic activity. Crow et al. also found that any gene mutation of the three subunits of the RNase H2 enzyme complex can lead to Aicardi-Goutières syndrome (AGS), in which the level of RNA: DNA hybrids is increased, suggesting that the formation of abnormal RNA: DNA hybrids contribute to AGS [139, 154]. In humans, the deficiency of RNase H2 can also cause other autoimmune diseases and cancers, such as systemic lupus erythematosus, skin cancer, and colorectal cancer [155].

Amyotrophic lateral sclerosis (ALS) is a common neurodegenerative disease. Type 4 amyotrophic lateral sclerosis (ALS4) is a rare autosomal dominant ALS that occurs in children or adolescents. Its clinical features are similar to ALS patients. In addition to limb weakness and muscle atrophy, it is also manifested in the slow progression of the disease [156, 157]. The pathogenesis of ALS4 is still unclear, but studies have shown that it is a neurological disease caused by mutations in the SETX gene. Furthermore, mutation of SETX also causes ataxia with oculomotor apraxia 2 (AOA-2), which is also a neurological disease [119, 156].

Genomic instability is an important hallmark of cancers. The non-programmed accumulation of the R-loop is associated with genomic instability. Therefore, there is a potential association between the R-loop and the development of cancers [22, 136]. BRCA1 and BRCA2 are two subtypes of breast cancer susceptibility genes. They are both tumor suppressor genes. Once mutations occur, they will increase the risk of cancers [137]. At the same time, studies have shown that BRCA1 and BRCA2 play an important role in preventing the accumulation of R-loop in the genome. Their mutations or deletions can lead to increased R-loop levels and DNA damage [83, 158]. In general, the accumulation of the R loop is one of the sources of genomic instability in cancer cells.

Recent studies have shown that RNA: DNA hybrids not only exist in the nucleus but also accumulate in the cytoplasm. When SETX or BRCA1 mutations cause nuclear R-loop dysregulation, the accumulation of hybrids in the cytoplasm is perceived by the immune receptors cGAS and TLR3, which in turn activates IRF3-mediated immune signals and apoptosis, and studies have shown that the cytoplasmic RNA: DNA hybrids derived from the R-loop is associated with human diseases, such as ataxia oculomotor apraxia type 2 (AOA2), Aicardi–Goutières syndrome and cancers [158, 159].

## Conclusions and perspectives

The regulation of the cell cycle is a complex process, which requires the interaction of many proteins, cytokines, and cell cycle signaling pathways to ensure its proper progression. The hallmark event of cell proliferation is DNA replication, which mainly occurs in the S phase. At this stage, when the transcription and the replication machinery share the same DNA template chain, it makes the collision between them occur more frequently and results in the generation of R-loop, which in turn affects the process of the replication fork. Although the physiological existence of the R-loop plays an important role in normal cell activities, its accumulation can threaten the stability of the genome, which is related to the occurrence and development of many diseases [160].

Under physiological conditions, the regulatory factors responsible for the formation and resolution of the R-loop need to work coordinately to maintain the steady-state balance of the R-loop in the cell cycle. At the same time, in response to R-loop-induced DNA damage, cell cycle checkpoint will delay cell cycle progression or induce cells to withdraw from the cell cycle, thereby avoiding gene deletion, mutation, or genomic instability.

Although R-loop is involved in a variety of disease processes, cell cycle-based dysregulation of the R-loop appears to have a greater impact on cancer. Cancer is a malignant disease characterized by unlimited proliferation and DNA replication, which improve the utilization of the genome as a template and increase the frequency of TRCs [161]. It has been reported that the occurrence of TRCs promotes the production of R-loop. Studies have shown that the accumulated R-loop in the nucleus to a certain extent can be released into the cytoplasm and perceived by immune receptors, which provoke anti-tumor immunity, that is the production of type I interferon (IFN-I) [158, 162]. In addition, genomes of cancer carry numerous somatic mutations, including the R-loop resolution-related gene, for example, BRCA1. It is clear that the deficiency of BRCA1 not only increases the risk of breast or ovarian cancers but also the level of R-loop. BRCA1 plays a key role in the decomposition of the R-loop, its deficiency causes the accumulation of R-loop in cancer cells, which in turn leads to subsequent genomic instability, replication stress, and loss of viability. Therefore, the R-loop may be a potential target for cancer therapy [163, 164]. Together, it is conceivable that the combination of TRCs and deficiency of BRCA1 can inevitably and synergistically elicit potent anti-tumor immunity, lead to cell cycle arrest, and even cell death, which may be a therapeutic vulnerability of cancer with defective BRCA1.

Among the aforementioned factors implicated in R-loop formation and resolution, only a few factors had been determined to exert their functions in specific cell cycle stages. Further work will be needed to identify more factors that have roles in each stage of cell cycle, especially in S phase. In human tumors, there are a large number of mutations [161], it is important to investigate how the function-altering mutations influence cell cycle progress when they affect a specific R-loop-related factor. Many R-loop-related factors exert their function in a context dependent manner [81], during the cell cycle progress, the context is changing constantly, it will be challenging to elucidate how these factors to adapt to the changing context to ensure the cell cycle progress smoothly.

## Data Availability

No datasets were generated or analysed during the current study.

## References

[1] Schwartz GK, Shah MA (2005). Targeting the cell cycle: a new approach to cancer therapy. J Clin Oncol.

[2] De Boeck J, Rombouts J, Gelens L (2021). A modular approach for modeling the cell cycle based on functional response curves. PLoS Comput Biol.

[3] Molina A (2022). Single-cell imaging of the cell cycle reveals CDC25B-induced heterogeneity of G1 phase length in neural progenitor cells. Development..

[4] Cooper S (2021). The Anti-G0 Manifesto: Should a problematic construct (G0) with no biological reality be removed from the cell cycle? Yes!. Bioessays.

[5] Chambard JC, Franchi A, Le Cam A, et al. Growth factor-stimulated protein phosphorylation in G0/G1-arrested fibroblasts. Two distinct classes of growth factors with potentiating effects [J]. J Biol Chem. 1983;258(3):1706-13. 6822530

[6] Houlard M (2011). DNA-RNA hybrids contribute to the replication dependent genomic instability induced by Omcg1 deficiency. Cell Cycle.

[7] Baek H, Park SU, Kim J (2023). Emerging role for R-loop formation in hepatocellular carcinoma. Genes Genomics..

[8] San Martin-Alonso M (2021). Harmful R-loops are prevented via different cell cycle-specific mechanisms. Nat Commun.

[9] Skourti-Stathaki K, Proudfoot NJ (2014). A double-edged sword: R loops as threats to genome integrity and powerful regulators of gene expression. Genes & Development.

[10] Castellano-Pozo M, Garcia-Muse T, Aguilera A (2012). R-loops cause replication impairment and genome instability during meiosis. EMBO Rep.

[11] Garcia-Muse T, Aguilera A (2019). R Loops: From Physiological to Pathological Roles. Cell.

[12] Matos DA (2020). ATR Protects the Genome against R Loops through a MUS81-Triggered Feedback Loop. Mol Cell..

[13] Tous C, Aguilera A (2007). Impairment of transcription elongation by R-loops in vitro. Biochem Biophys Res Commun.

[14] Dutta D (2011). Linking RNA polymerase backtracking to genome instability in E coli. Cell.

[15] Chao HX (2017). Orchestration of DNA Damage Checkpoint Dynamics across the Human Cell Cycle. Cell Syst..

[16] Achar YJ, Foiani M (2017). Coordinating Replication with Transcription. Adv Exp Med Biol.

[17] Salas-Armenteros I (2017). Human THO-Sin3A interaction reveals new mechanisms to prevent R-loops that cause genome instability. EMBO J.

[18] Dou P (2020). C1orf109L binding DHX9 promotes DNA damage depended on the R-loop accumulation and enhances camptothecin chemosensitivity. Cell Prolif.

[19] Lockhart A (2019). RNase H1 and H2 Are Differentially Regulated to Process RNA-DNA Hybrids. Cell Rep..

[20] Petermann E, Lan L, Zou L (2022). Sources, resolution and physiological relevance of R-loops and RNA-DNA hybrids. Nat Rev Mol Cell Biol.

[21] Fletcher CE (2022). A non-coding RNA balancing act: miR-346-induced DNA damage is limited by the long non-coding RNA NORAD in prostate cancer. Mol Cancer.

[22] Richard P, Manley JL (2017). R Loops and Links to Human Disease. J Mol Biol.

[23] Schafer KA (1998). The cell cycle: a review. Vet Pathol.

[24] Wang Z (2022). Cell Cycle Progression and Synchronization: An Overview. Methods Mol Biol.

[25] Uzbekov R, Prigent C (2022). A Journey through Time on the Discovery of Cell Cycle Regulation. Cells.

[26] Matthews HK, Bertoli C, de Bruin RAM (2022). Cell cycle control in cancer. Nat Rev Mol Cell Biol.

[27] Gerard C (2015). Cell cycle control by a minimal Cdk network. PLoS Comput Biol.

[28] Satyanarayana A, Kaldis P (2009). Mammalian cell-cycle regulation: several Cdks, numerous cyclins and diverse compensatory mechanisms. Oncogene.

[29] Barnum KJ, O'Connell MJ (2014). Cell cycle regulation by checkpoints. Methods Mol Biol.

[30] Marnef A, Legube G (2021). R-loops as Janus-faced modulators of DNA repair. Nat Cell Biol.

[31] Wongsurawat T (2020). R-loop-forming Sequences Analysis in Thousands of Viral Genomes Identify A New Common Element in Herpesviruses. Sci Rep.

[32] Malig M (2020). Ultra-deep Coverage Single-molecule R-loop Footprinting Reveals Principles of R-loop Formation. J Mol Biol.

[33] Santos-Pereira JM, Aguilera A (2015). R loops: new modulators of genome dynamics and function. Nat Rev Genet.

[34] Kim N, Jinks-Robertson S (2012). Transcription as a source of genome instability. Nat Rev Genet.

[35] Chakraborty P (2020). New insight into the biology of R-loops. Mutat Res.

[36] Daube SS, von Hippel PH (1994). RNA displacement pathways during transcription from synthetic RNA-DNA bubble duplexes. Biochemistry.

[37] Belotserkovskii BP, Hanawalt PC (2022). Topology and kinetics of R-loop formation. Biophys J.

[38] Chakraborty P, Huang JTJ, Hiom K (2018). DHX9 helicase promotes R-loop formation in cells with impaired RNA splicing. Nat Commun.

[39] Ginno PA (2012). R-loop formation is a distinctive characteristic of unmethylated human CpG island promoters. Mol Cell.

[40] Ginno PA (2013). GC skew at the 5' and 3' ends of human genes links R-loop formation to epigenetic regulation and transcription termination. Genome Res.

[41] Mirkin EV, Mirkin SM (2007). Replication Fork Stalling at Natural Impediments. Microbiology and Molecular Biology Reviews.

[42] Deng Z, Wang Z, Lieberman PM (2012). Telomeres and viruses: common themes of genome maintenance. Front Oncol..

[43] Reddy K (2011). Determinants of R-loop formation at convergent bidirectionally transcribed trinucleotide repeats. Nucleic Acids Res.

[44] Loomis EW (2014). Transcription-associated R-loop formation across the human FMR1 CGG-repeat region. PLoS Genet.

[45] Kang HJ (2021). Thrap3 promotes R-loop resolution via interaction with methylated DDX5. Exp Mol Med.

[46] Edwards DS (2020). BRD4 Prevents R-Loop Formation and Transcription-Replication Conflicts by Ensuring Efficient Transcription Elongation. Cell Rep.

[47] Li X, Manley JL (2005). Inactivation of the SR protein splicing factor ASF/SF2 results in genomic instability. Cell.

[48] Balk B (2013). Telomeric RNA-DNA hybrids affect telomere-length dynamics and senescence. Nat Struct Mol Biol.

[49] Arora R, Azzalin CM (2015). Telomere elongation chooses TERRA ALTernatives. RNA Biol.

[50] Graf M (2017). Telomere Length Determines TERRA and R-Loop Regulation through the Cell Cycle. Cell..

[51] Wahba L (2011). RNase H and multiple RNA biogenesis factors cooperate to prevent RNA:DNA hybrids from generating genome instability. Mol Cell.

[52] Lima WF (2016). Viable RNaseH1 knockout mice show RNaseH1 is essential for R loop processing, mitochondrial and liver function. Nucleic Acids Res.

[53] Cerritelli SM, Crouch RJ (2009). Ribonuclease H: the enzymes in eukaryotes. FEBS J.

[54] Perez-Calero C (2020). UAP56/DDX39B is a major cotranscriptional RNA-DNA helicase that unwinds harmful R loops genome-wide. Genes Dev.

[55] San Martin Alonso M, Noordermeer SM (2021). Untangling the crosstalk between BRCA1 and R-loops during DNA repair. Nucleic Acids Res.

[56] Promonet A (2020). Topoisomerase 1 prevents replication stress at R-loop-enriched transcription termination sites. Nat Commun.

[57] Sarni D (2022). Topoisomerase 1-dependent R-loop deficiency drives accelerated replication and genomic instability. Cell Rep.

[58] Garcia-Rubio ML (2015). The Fanconi Anemia Pathway Protects Genome Integrity from R-loops. PLoS Genet.

[59] Yarden RI (2002). BRCA1 regulates the G2/M checkpoint by activating Chk1 kinase upon DNA damage. Nat Genet.

[60] Hatchi E (2015). BRCA1 recruitment to transcriptional pause sites is required for R-loop-driven DNA damage repair. Mol Cell.

[61] Cargill M, Venkataraman R, Lee S (2021). DEAD-Box RNA Helicases and Genome Stability. Genes (Basel)..

[62] Luna R (2019). The THO Complex as a Paradigm for the Prevention of Cotranscriptional R-Loops. Cold Spring Harb Symp Quant Biol.

[63] Bayona-Feliu A (2021). The SWI/SNF chromatin remodeling complex helps resolve R-loop-mediated transcription-replication conflicts. Nat Genet.

[64] Davó-Martínez C (2023). Different SWI/SNF complexes coordinately promote R-loop- and RAD52-dependent transcription-coupled homologous recombination. Nucleic Acids Res..

[65] Kang HJ (2021). TonEBP recognizes R-loops and initiates m6A RNA methylation for R-loop resolution. Nucleic Acids Res.

[66] Abakir A (2020). N(6)-methyladenosine regulates the stability of RNA:DNA hybrids in human cells. Nat Genet.

[67] Laverde EE (2022). Flap Endonuclease 1 Endonucleolytically Processes RNA to Resolve R-Loops through DNA Base Excision Repair. Genes (Basel).

[68] Park K (2021). Aicardi-Goutieres syndrome-associated gene SAMHD1 preserves genome integrity by preventing R-loop formation at transcription-replication conflict regions. PLoS Genet.

[69] Shiromoto Y (2021). ADAR1 RNA editing enzyme regulates R-loop formation and genome stability at telomeres in cancer cells. Nat Commun.

[70] Cui H (2022). ADAR1 Prevents R-loop Accumulation-Driven ATR Pathway Activation in Ovarian Cancer. J Cancer.

[71] Lin WL (2022). DDX18 prevents R-loop-induced DNA damage and genome instability via PARP-1. Cell Rep.

[72] Cohen S (2018). Senataxin resolves RNA:DNA hybrids forming at DNA double-strand breaks to prevent translocations. Nat Commun.

[73] Boleslavska B (2022). DDX17 helicase promotes resolution of R-loop-mediated transcription-replication conflicts in human cells. Nucleic Acids Res.

[74] Hodroj D (2017). An ATR-dependent function for the Ddx19 RNA helicase in nuclear R-loop metabolism. EMBO J.

[75] Yu Z (2020). DDX5 resolves R-loops at DNA double-strand breaks to promote DNA repair and avoid chromosomal deletions. NAR Cancer..

[76] Li L (2023). DEAD Box 1 Facilitates Removal of RNA and Homologous Recombination at DNA Double-Strand Breaks. Molecular and Cellular Biology.

[77] Mosler T (2021). R-loop proximity proteomics identifies a role of DDX41 in transcription-associated genomic instability. Nat Commun.

[78] Song C (2017). SIRT7 and the DEAD-box helicase DDX21 cooperate to resolve genomic R loops and safeguard genome stability. Genes & Development.

[79] Sakasai R (2017). Aquarius is required for proper CtIP expression and homologous recombination repair. Scientific Reports.

[80] Tan J (2020). Resolution of ROS-induced G-quadruplexes and R-loops at transcriptionally active sites is dependent on BLM helicase. FEBS Letters.

[81] Brickner JR, Garzon JL, Cimprich KA (2022). Walking a tightrope: The complex balancing act of R-loops in genome stability. Molecular Cell.

[82] Svikovic S (2019). R-loop formation during S phase is restricted by PrimPol-mediated repriming. EMBO J..

[83] Shivji MKK (2018). BRCA2 Regulates Transcription Elongation by RNA Polymerase II to Prevent R-Loop Accumulation. Cell Reports.

[84] Chang EY (2019). MRE11-RAD50-NBS1 promotes Fanconi Anemia R-loop suppression at transcription-replication conflicts. Nat Commun.

[85] Goulielmaki E (2021). The splicing factor XAB2 interacts with ERCC1-XPF and XPG for R-loop processing. Nature Communications..

[86] Makharashvili N (2018). Sae2/CtIP prevents R-loop accumulation in eukaryotic cells. ELife.

[87] Yeo AJ (2014). R-loops in proliferating cells but not in the brain: implications for AOA2 and other autosomal recessive ataxias. PLoS One.

[88] Feretzaki M (2020). RAD51-dependent recruitment of TERRA lncRNA to telomeres through R-loops. Nature.

[89] Mazina OM (2020). Replication protein A binds RNA and promotes R-loop formation. Journal of Biological Chemistry.

[90] Nojima T (2018). Deregulated Expression of Mammalian lncRNA through Loss of SPT6 Induces R-Loop Formation, Replication Stress, and Cellular Senescence. Molecular Cell.

[91] Teloni F (2019). Efficient Pre-mRNA Cleavage Prevents Replication-Stress-Associated Genome Instability. Molecular Cell.

[92] Dang TT, Morales JC (2020). XRN2 Links RNA:DNA Hybrid Resolution to Double Strand Break Repair Pathway Choice. Cancers..

[93] Syuzo Kaneko CC (2007). Aaron J Shatkin, James L Manley, Human capping enzyme promotes formation of transcriptional R loops in vitro. Proc Natl Acad Sci U S A.

[94] Prendergast L (2020). Resolution of R-loops by INO80 promotes DNA replication and maintains cancer cell proliferation and viability. Nat Commun.

[95] Herrera-Moyano E (2014). The yeast and human FACT chromatin-reorganizing complexes solve R-loop-mediated transcription–replication conflicts. Genes & Development.

[96] Jae Jin Kim, S.Y.L., Fade Gong,Anna M Battenhouse,Daniel R Boutz,Aarti Bashyal,Samantha T Refvik,Cheng-Ming Chiang,Blerta Xhemalce,Tanya T Paull,Jennifer S Brodbelt,Edward M Marcotte,Kyle M Miller, systematic bromodomain protein screens identify homologous recombination and r-loop suppression Genes Dev . 2019. 10.1101/gad.331231.119 PMC6942044 31753913

[97] Leng F (2017). The monoclonal S9.6 antibody exhibits highly variable binding affinities towards different R-loop sequences. Plos One.

[98] Barroso S (2019). The DNA damage response acts as a safeguard against harmful DNA-RNA hybrids of different origins. EMBO Rep.

[99] Wei X (1998). Segregation of transcription and replication sites into higher order domains. Science.

[100] Groelly FJ (2022). Mitotic DNA synthesis is caused by transcription-replication conflicts in BRCA2-deficient cells. Mol Cell..

[101] Marabitti V (2022). R-Loop-Associated Genomic Instability and Implication of WRN and WRNIP1. Int J Mol Sci.

[102] Hamperl S, et al. Transcription-Replication Conflict Orientation Modulates R-Loop Levels and Activates Distinct DNA Damage Responses. Cell. 2017;170(4):774–786 e19. 10.1016/j.cell.2017.07.043 PMC5570545 28802045

[103] Lang KS (2017). Replication-Transcription Conflicts Generate R-Loops that Orchestrate Bacterial Stress Survival and Pathogenesis. Cell.

[104] Garcia-Muse T, Aguilera A (2016). Transcription-replication conflicts: how they occur and how they are resolved. Nat Rev Mol Cell Biol.

[105] Rocha EP (2008). The organization of the bacterial genome. Annu Rev Genet.

[106] Kohler A, Hurt E (2007). Exporting RNA from the nucleus to the cytoplasm. Nat Rev Mol Cell Biol.

[107] Sogo JM, Lopes M, Foiani M (2002). Fork reversal and ssDNA accumulation at stalled replication forks owing to checkpoint defects. Science.

[108] Gomez-Gonzalez B, Felipe-Abrio I, Aguilera A (2009). The S-phase checkpoint is required to respond to R-loops accumulated in THO mutants. Mol Cell Biol.

[109] Vijayraghavan S, Tsai FL, Schwacha A (2016). A Checkpoint-Related Function of the MCM Replicative Helicase Is Required to Avert Accumulation of RNA:DNA Hybrids during S-phase and Ensuing DSBs during G2/M. PLoS Genet.

[110] Tsai F-L (2023). Mcm2-7 Is an Active Player in the DNA Replication Checkpoint Signaling Cascade via Proposed Modulation of Its DNA Gate. Molecular and Cellular Biology.

[111] Rubin SM, Sage J, Skotheim JM (2020). Integrating Old and New Paradigms of G1/S Control. Molecular Cell.

[112] Lisby M, Rothstein R, Mortensen UH (2001). Rad52 forms DNA repair and recombination centers during S phase. Proc Natl Acad Sci U S A.

[113] Strasser K (2002). TREX is a conserved complex coupling transcription with messenger RNA export. Nature.

[114] Polenkowski M (2023). THOC5 complexes with DDX5, DDX17, and CDK12 to regulate R loop structures and transcription elongation rate. iScience.

[115] Dominguez-Sanchez MS (2011). Genome instability and transcription elongation impairment in human cells depleted of THO/TREX. PLoS Genet.

[116] Aguilera A (2002). The connection between transcription and genomic instability. EMBO J.

[117] Wellinger RE, Prado F, Aguilera A (2006). Replication fork progression is impaired by transcription in hyperrecombinant yeast cells lacking a functional THO complex. Mol Cell Biol.

[118] Gomez-Gonzalez B, Aguilera A (2019). Transcription-mediated replication hindrance: a major driver of genome instability. Genes Dev.

[119] Moreira MC (2004). Senataxin, the ortholog of a yeast RNA helicase, is mutant in ataxia-ocular apraxia 2. Nat Genet.

[120] Groh M (2017). Senataxin: Genome Guardian at the Interface of Transcription and Neurodegeneration. J Mol Biol.

[121] Hasanova Z (2023). Human senataxin is a bona fide R-loop resolving enzyme and transcription termination factor. Nucleic Acids Res..

[122] Skourti-Stathaki K, Nicholas Proudfoot J, Gromak N (2011). Human Senataxin Resolves RNA/DNA Hybrids Formed at Transcriptional Pause Sites to Promote Xrn2-Dependent Termination. Molecular Cell.

[123] Mischo HE, et al. Cell-Cycle Modulation of Transcription Termination Factor Sen1. Mol Cell. 2018;70(2):312–326 e317. 10.1016/j.molcel.2018.03.010 PMC5919780 29656924

[124] Alzu A (2012). Senataxin associates with replication forks to protect fork integrity across RNA-polymerase-II-transcribed genes. Cell.

[125] Kannan A, et al. ZPR1 prevents R-loop accumulation, upregulates SMN2 expression and rescues spinal muscular atrophy. Brain. 2020;143(1):69–93. 10.1093/brain/awz373 PMC6935747 31828288

[126] Aiello U (2022). Sen1 is a key regulator of transcription-driven conflicts. Mol Cell.

[127] Ramirez, P., et al., R-Loop Analysis by Dot-Blot. J Vis Exp, 2021(167) 10.3791/62069 PMC8506650 33554969

[128] Williams JS, Gehle DB, Kunkel TA (2017). The role of RNase H2 in processing ribonucleotides incorporated during DNA replication. DNA Repair.

[129] O’Connell K, Jinks-Robertson S, Petes TD (2015). Elevated Genome-Wide Instability in Yeast Mutants Lacking RNase H Activity. Genetics.

[130] Cerritelli SM, Sakhuja K, Crouch RJ (2022). RNase H1, the Gold Standard for R-Loop Detection. Methods Mol Biol.

[131] Arudchandran A (2000). The absence of ribonuclease H1 or H2 alters the sensitivity of Saccharomyces cerevisiae to hydroxyurea, caffeine and ethyl methanesulphonate: implications for roles of RNases H in DNA replication and repair. Genes Cells.

[132] Zimmer AD, Koshland D (2016). Differential roles of the RNases H in preventing chromosome instability. Proc Natl Acad Sci U S A.

[133] Parajuli S (2017). Human ribonuclease H1 resolves R-loops and thereby enables progression of the DNA replication fork. J Biol Chem.

[134] Nguyen HD (2017). Functions of Replication Protein A as a Sensor of R Loops and a Regulator of RNaseH1. Molecular Cell.

[135] Li M, Klungland A (2020). Modifications and interactions at the R-loop. DNA Repair (Amst).

[136] Groh M, Gromak N (2014). Out of balance: R-loops in human disease. PLoS Genet.

[137] Saleem M (2018). The BRCA1 and BRCA2 Genes in Early-Onset Breast Cancer Patients. Cancer Biol Adv Treat.

[138] Bhatia V (2014). BRCA2 prevents R-loop accumulation and associates with TREX-2 mRNA export factor PCID2. Nature.

[139] Lim YW (2015). Genome-wide DNA hypomethylation and RNA:DNA hybrid accumulation in Aicardi-Goutières syndrome. ELife.

[140] mutation in senataxin alters the mechanism of r loop resolution in amyotro source brain 10.1093/brain/awab464 PMC9536298 35045161

[141] Gordenin DA (2020). TDP-43 mutations link Amyotrophic Lateral Sclerosis with R-loop homeostasis and R loop-mediated DNA damage. PLOS Genet..

[142] Becherel OJ (2015). A new model to study neurodegeneration in ataxia oculomotor apraxia type 2. Human Molecular Genetics.

[143] Cai S (2020). Knockdown of THOC1 reduces the proliferation of hepatocellular carcinoma and increases the sensitivity to cisplatin. J Exp Clin Cancer Res.

[144] Zhang H (2021). MTA2 triggered R-loop trans-regulates BDH1-mediated β-hydroxybutyrylation and potentiates propagation of hepatocellular carcinoma stem cells. Signal Transduct Target Ther..

[145] Ye BJ (2021). PARP1-mediated PARylation of TonEBP prevents R-loop–associated DNA damage. DNA Repair.

[146] Chernikova SB (2012). Deficiency in Mammalian Histone H2B Ubiquitin Ligase Bre1 (Rnf20/Rnf40) Leads to Replication Stress and Chromosomal Instability. Cancer Research.

[147] Farg MA (2017). The DNA damage response (DDR) is induced by the C9orf72 repeat expansion in amyotrophic lateral sclerosis. Human Molecular Genetics.

[148] Aguilera A (2014). R-loops Associated with Triplet Repeat Expansions Promote Gene Silencing in Friedreich Ataxia and Fragile X Syndrome. PLoS Genet..

[149] Garcia-Moreno H (2023). Neurological disease in xeroderma pigmentosum: prospective cohort study of its features and progression. Brain.

[150] Weinreb JT (2021). Excessive R-loops trigger an inflammatory cascade leading to increased HSPC production. Developmental Cell.

[151] Sagie S, et al. Telomeres in ICF syndrome cells are vulnerable to DNA damage due to elevated DNA RNA hybrids. Nat Commun. 2017;8(1):14015-26. 10.1038/ncomms14015 PMC5286223 28117327

[152] Xu K (2021). Therapeutic Development for CGG Repeat Expansion-Associated Neurodegeneration. Frontiers in Cellular Neuroscience..

[153] Lee-Kirsch MA, Wolf C, Günther C. Aicardi-Goutières syndrome: a model disease for systemic autoimmunity. Clinical and Experimental Immunology. 2014;175(1):17–24. 10.1111/cei.12160 PMC3898550 23786362

[154] Crow YJ (2006). Mutations in genes encoding ribonuclease H2 subunits cause Aicardi-Goutieres syndrome and mimic congenital viral brain infection. Nat Genet.

[155] Cerritelli SM, El Hage A (2020). RNases H1 and H2: guardians of the stability of the nuclear genome when supply of dNTPs is limiting for DNA synthesis. Curr Genet.

[156] Chen YZ (2004). DNA/RNA helicase gene mutations in a form of juvenile amyotrophic lateral sclerosis (ALS4). Am J Hum Genet.

[157] Bennett CL (2018). Senataxin mutations elicit motor neuron degeneration phenotypes and yield TDP-43 mislocalization in ALS4 mice and human patients. Acta Neuropathologica.

[158] Crossley, M.P., et al., R-loop-derived cytoplasmic RNA-DNA hybrids activate an immune response. Nature, 2022. 10.1038/s41586-022-05545-9 PMC9949885 36544021

[159] An Autoantibody Subset Can Be Used for SCLC Early Detection. Cancer Discov, 2023;13(3):526. 10.1158/2159-8290.CD-RW2023-009 36661372

[160] Allison DF, Wang GG (2019). R-loops: formation, function, and relevance to cell stress. Cell Stress.

[161] Hanahan D, Robert A (2011). Weinberg, Hallmarks of Cancer: The Next Generation. Cell.

[162] Alexandrov Ludmil B (2013). Deciphering Signatures of Mutational Processes Operative in Human Cancer. Cell Reports.

[163] Jaiswal AS (2023). TATDN2 resolution of R-loops is required for survival of BRCA1-mutant cancer cells. Nucleic Acids Research.

[164] S Parasvi , A.H. Patel, Hakem Razqallah (2021). RNF168 regulates R-loop resolution and genomic stability in BRCA1/2-deficient tumors. J Clin Invest.

